# The effect of the algal microbiome on industrial production of microalgae

**DOI:** 10.1111/1751-7915.13296

**Published:** 2018-07-05

**Authors:** Jie Lian, Rene H. Wijffels, Hauke Smidt, Detmer Sipkema

**Affiliations:** ^1^ Laboratory of Microbiology Wageningen University & Research Stippeneng 4 6708 WE Wageningen The Netherlands; ^2^ Bioprocess Engineering Group, AlgaePARC Wageningen University & Research PO Box 16 6700 AA Wageningen The Netherlands; ^3^ Faculty of Biosciences and Aquaculture Nord University N‐8049 Bodø Norway

## Abstract

Microbes are ubiquitously distributed, and they are also present in algae production systems. The algal microbiome is a pivotal part of the alga holobiont and has a key role in modulating algal populations in nature. However, there is a lack of knowledge on the role of bacteria in artificial systems ranging from laboratory flasks to industrial ponds. Coexisting microorganisms, and predominantly bacteria, are often regarded as contaminants in algal research, but recent studies manifested that many algal symbionts not only promote algal growth but also offer advantages in downstream processing. Because of the high expectations for microalgae in a bio‐based economy, better understanding of benefits and risks of algal–microbial associations is important for the algae industry. Reducing production cost may be through applying specific bacteria to enhance algae growth at large scale as well as through preventing the growth of a broad spectrum of algal pathogens. In this review, we highlight the latest studies of algae–microbial interactions and their underlying mechanisms, discuss advantages of large‐scale algal–bacterial cocultivation and extend such knowledge to a broad range of biotechnological applications.

## Introduction

During the last forty years, efforts have been undertaken to realize the high potential of algal products for industrial applications. Algae have been widely recognized for their capacity to produce polysaccharides, lipids, pigments and other valuable compounds in significant amounts (Wijffels and Barbosa, 2010). Algae are used for producing healthy food and food supplements, and as an ingredient in aquaculture, animal feed and as soil biofertilizer (Sharma *et al*., 2011; Shields and Lupatsch, 2012).

Most algae, if not all, live in symbiosis with multiple associated microorganisms throughout their lifespan (Dittami *et al*., 2014). In many cases, attempts to remove bacteria or fungi from microalgae have failed. Even in cases where such attempts were successful, microbiota‐deprived algae usually exhibited poorer growth or aberrant phenotypes compared to the original strains, which indicates that the association between algae and other microorganisms is important for their existence (Hom *et al*., 2015).

Algae are known to release dissolved organic matter or signalling molecules to nurture specific bacterial communities in the phycosphere (Amin *et al*., 2012). Close interactions in the phycosphere influence algal evolution and ecology in various ways. First of all, algae such as the diatoms *Phaeodactylum tricornutum* and *Thalassiasira pseudonana* have been shown to have acquired hundreds of genes predicted to be involved in nitrogen and organic carbon utilization, cell wall assembly, DNA recombination and the ornithine‐urea cycle from co‐occurring bacteria during more than 200 million years (Bowler *et al*., 2008). Second, bacteria synthesize important compounds for algal growth stimulation, spore germination, morphogenesis and pathogen resistance (Amin *et al*., 2012, 2015; Ramanan *et al*., 2016). These compounds include micronutrients, siderophores, growth stimulants and antibiotics (Bruhn *et al*., 2007; Amin *et al*., 2009; Seyedsayamdost *et al*., 2011; Wahl *et al*., 2012; Natrah *et al*., 2013; Danchin and Braham, 2017). In addition, symbiotic microorganisms help their algal hosts to cope with changing environmental conditions (Xie *et al*., 2013a; Dittami *et al*., 2016).

On the other hand, many microbes have been reported to negatively affect algal growth (Le Chevanton *et al*., 2013; Kim *et al*., 2014) and constitute big constraints for translating laboratory experiments to industrial practice. Unlike conventional microbial fermentation, large‐scale algal cultivation is driven by light and mostly operated in fully exposed open ponds for microalgae and in open sea for macroalgae. However, open ponds are more susceptible to biological contaminations, such as viruses, predators/grazers and parasites of various sources (Carney and Lane, 2014). Therefore, stable production of algae in open systems is only possible when contaminants and infections are well studied so that monitoring and contingency measures can be implemented (Mendes and Vermelho, 2013).

Apart from playing a role in enhancing microalgae production, associated bacteria can help the algae to perform more complex tasks with diverse applications. For instance, algae and bacteria cooperate in faster and more efficient removal of organic and inorganic waste and hazardous substances in wastewater treatment (Su *et al*., 2012; Luo *et al*., 2014; Cavaliere *et al*., 2017). In turn, bacterial and viral pathogens are able to weaken or decompose the algal cell wall, which is a crucial step in algal‐based extraction of chemicals and could also be explored to tackle frequently occurring harmful algae blooms at an early stage of the bloom (Wilson *et al*., 2002; Chen *et al*., 2014). Furthermore, proteins or secondary metabolites of algicidal bacteria are potential biological agents in algal biomass harvest and cell disruption prior to biorefinery (Lenneman *et al*., 2014).

**Figure 1:**
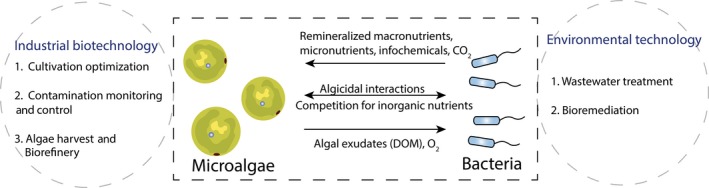
Potential applications of algal–bacterial interactions in industrial biotechnology and environmental biotechnology. DOM is dissolved organic matter.

The aim of this review was to provide an overview of both beneficial and antagonistic algal–microbial interactions in natural and artificial systems, as well as to provide new perspectives about how to utilize such knowledge in algal biotechnology (Fig. 1).

## Alga‐associated bacteria in algae production systems

Although next‐generation sequencing (NGS) has led to an explosion of microbial diversity studies in microbial ecology research, only a limited number of studies have been published on NGS‐based microbiota analysis in the context of microalgal production systems. In fact, most knowledge of alga–bacteria communities in applied settings come from wastewater treatment studies (García *et al*., 2017; Sun *et al*., 2018; Yang *et al*., 2018). However, those systems are too different to microalgae production systems due to the presence of high concentrations of organic and inorganic material to expect a large overlap in microbial communities in wastewater treatment systems and algae production facilities. For that reason, wastewater treatment with algae–bacteria consortia is treated separately in Section Nutrient provision. The molecular survey of bacterial diversity in three cultures (*Nannochloropsis salina* from a raceway pond and a closed photobioreactor, respectively, and *Botryococcus braunii* from laboratory flasks) (Carney *et al*., 2016; Sambles *et al*., 2017; Fulbright *et al*., 2018) and one biofilm sample from an outdoor photobioreactor (mixture of *Chlorella vulgaris* and *Scenedesmus obliquus*) (Krohn‐Molt *et al*., 2013) revealed that *Deltaproteobacteria* and *Gammaproteobacteria* in raceway pond and *Alphaproteobacteria* and *Bacteroidetes* in closed bioreactor were dominant in *N. salina,* whereas *Gammaproteobacteria*,* Betaproteobacteria* and *Firmicutes* were the most prominent phyla in *B. braunii*. *Alphaproteobacteria*,* Bacteroidetes*,* Betaproteobacteria* and *Gammaproteobacteri*a made up nearly three‐quarters of the biofilm bacterial community. Based on this limited number of studies, *Proteobacteria*, and *Gammaproteobacteria,* in particular, are found associated with cultured microalgae. *Cytophagales* and *Flavobacteriales* were the only two common bacterial orders among four studies. Several other taxa such as *Pseudomonadales*,* Burkholderiales*,* Caulobacterales* and *Rhodobacterales* were shared between either two studies. Our limited knowledge of bacterial communities associated with microalgae that is based on cultivation‐independent studies currently prevents general statements about bacteria that are frequently found associated with microalgae, but finding correlations between algae and associated bacteria will be a good starting point for coming up with hypotheses on functional relationships. Therefore, more studies of bacterial communities found in microalgae bioreactors are urgently needed to obtain a clearer view on the species and genera that are commonly associated with algae.

## Beneficial roles of bacteria

Although for most of the bacteria detected in microalgae production systems it is not known if/how they interact with the microalgae, recent observations have demonstrated that mutualistic algal–bacterial interactions are prevalent (Seymour *et al*., 2017). Multiple bacteria have been tested in cocultivation to evaluate the effects on the growth of microalgae (Le Chevanton *et al*., 2013; Sison‐Mangus *et al*., 2014; Biondi *et al*., 2017), or more specifically looked at the exchange of metabolites and how bacteria may lead to more robust algal cultures that can better withstand environmental perturbations.

### Alga‐associated bacteria that enhance algal growth

Using either axenic or non‐axenic algal cultures, a number of different bacteria ranging from specific isolates to microbial communities present in tap water have been evaluated for their effects on microalgae growth (Table 1). The best studied algae with respect to associated bacteria are members of the genus *Chlorella* (Table 1). Bacteria that have been shown to be beneficial to *Chlorella vulgaris* include members of the genera *Bacillus, Flavobacterium, Rhizobium, Hyphomonas* and *Sphingomonas*. *Bacillus pumilus* ES4 was shown to promote *Chlorella vulgaris* growth by providing fixed atmospheric nitrogen (Hernandez *et al*., 2009). In another study when *Chlorella vulgaris* was cultivated with four different bacteria, maximum algal growth rate and final cell mass increased from 0.22 day^−1^ to 0.47 day^−1^ and from 1.3 g/l to 3.31 g/l respectively (Table 1). This increased growth was furthermore accompanied by a slight rise in algal lipid content from 22.4% to 28% (Cho *et al*., 2014).

**Table 1:** Impact of added bacteria on microalgae growth

Microalga	Added bacteria	Effect	Methodology to prepare axenic algae	Reference
*Chlorella vulgaris*	*Bacillus pumilus*	Final cell density increased by 150% in N‐free medium	Axenic but method not mentioned	Hernandez *et al*. (2009)
*Chlorella vulgaris*	*Flavobacterium* sp., *Rhizobium* sp, *Hyphomonas* sp, *Sphingomonas* sp.	Cell density increased by more than 100%	Ultrasonication, fluorescence‐activated cell sorter and micropicking	Cho *et al*. (2014)
*Chlorella vulgaris*	*Rhizobium* sp.	Cell count increased 72%, and growth rate increased by 11%	Not axenic	Kim *et al*. (2014)
*Chlorella vulgaris*	Multiple bacteria from tap water	Higher growth rate	Not axenic	Lakaniemi *et al*. (2012)
*Chlorella ellipsoidea*	*Brevundimonas* sp.	Algal cell density increased three times after seven days	Serial streaking	Park *et al*. (2007)
*Chlorella sorokiniana* IAM C‐212	*Microbacterium trichotecenolyticum*	Growth rate increased 16%	Streptomycin, gentamicin, penicillin G, vancomycin and pimaricin	Watanabe *et al*. (2005)
*Dunaliella* sp. SAG 19.3	*Alteromonas* sp. and *Muricauda* sp.	Biomass enhanced by 22%, 26%	Ampicillin, gentamicin, kanamycin and neomycin	Le Chevanton *et al*. (2013)
*Botryococcus braunii*	BOTRYCO‐2	Grow faster and biomass enhanced by 80%	Ampicillin	Tanabe *et al*. (2015)
*Lobomonas rostrata*	*Mesorhizobium loti*	Providing vitamin B_12_	Axenic but method not mentioned	Grant *et al*. (2014)
*Scrippsiella trochoidea*	*Marinobacter* sp. strain DG879	Cell density increased over 6%	Streptomycin	Amin *et al*. (2009)
*Thalassiosira rotula*	*Roseobacter* sp. and *Hyphomonas* sp.	Earlier start of growth and higher algal cell numbers	Axenic but method not mentioned	Grossart and Simon (2007)
*Phaeodactylum tricornutum* Utex 646	*Alphaproteobacteria* sp. strain 29	Cell density increased up to 55%	Axenic but method not mentioned	Bruckner *et al*. (2011)

Similar to *Chlorella,* also for other green algae, such as those belonging to the genera *Dunaliella*,* Botryococcus* and *Lobomonas* beneficial effects were observed when adding specific bacterial partners to axenic cultures (Table 1). Biomass accumulation of *Botryococcus braunii* was almost doubled compared with that of axenic cultures (Tanabe *et al*., 2015). Similarly, biomass production of *Dunaliella* sp. SAG 19.3 increased by 22% and 26% when cocultivated with *Alteromonas* sp. or *Muricauda* sp. respectively (Le Chevanton *et al*., 2013). Furthermore, it could be shown that the vitamin B_12_ synthesizing bacterium *Mesorhizobium loti* is indispensable for the survival of *Lobomonas rostrata* under conditions where the alga is cultivated without exogenous vitamin B_12_ (Grant *et al*., 2014). Two diatoms and one dinoflagellate were all observed to benefit from coexisting bacteria (Table 1), as indicated by either higher cell numbers or a faster growth rate of the algae. The strongest stimulation of growth was reported for *Phaeodactylum tricornutum* in the presence of the *Alphaproteobacterium* strain 29, as demonstrated by a 55% rise in cell density (Bruckner *et al*., 2011).

### Microbial‐associated salinity acclimation and thermal tolerance

Salinity is the major environmental factor that determines the distribution and performance of marine algae (Olsenz, 2011; Ras *et al*., 2013). Interestingly, in addition to their more direct ecophysiological roles, bacteria can also present a gene reservoir for algal evolution towards adaptation to different environmental conditions via horizontal gene transfer. The green alga *Picochlorum* sp. SENEW3 has a wide salt tolerance from at least 0.35% to 10.8% (Wang *et al*., 2014). Compared to its less halotolerant sisters, the genome of the salt‐tolerant strain was found to contain a suite of additional functional genes, 24 of which were derived from bacterial sources and were functional in response to salt stress (Foflonker *et al*., 2015). Although not a microalga, it is interesting to note that the transition of the brown macroalga *Ectocarpus* sp. strain 371 from seawater to freshwater medium greatly depended on the associated bacterial community. Strain 371 is a small filamentous brown alga with broad range salinity tolerance that is mediated by adjusting cell wall structure and metabolism (Charrier *et al*., 2008; Ritter *et al*., 2010; Tonon *et al*., 2011). Cultures deprived of associated microbes were unable to survive a salinity change, while this capability could be restored by restoring their microbiota (Dittami *et al*., 2016).

Temperature is another important factor affecting growth and survival of algae (Ras *et al*., 2013). This is relevant as industrially grown algal strains in shallow production ponds or flat panel bioreactors are exposed to considerable temperature fluctuations. The unicellular microalga *Chlamydomanas reinhardtii* grows best at a temperature between 20–32°C (Schroda, 2004). The direct transfer of *C. reinhardtii* from an optimum (25°C) to a rather high temperature (45°C) results in chlorosis and cell death, which are caused by the repression of cobalamin‐independent methionine synthase during heat stress. Through adding exogenous cobalamin or co‐cultures of the alga with a cobalamin‐producing bacterium (*Sinorhizobium meliloti*), cobalamin‐dependent methionine synthase mediated methionine biosynthesis could be reactivated, thereby preventing death of algal cell (Xie *et al*., 2013a).

Hence, a better understanding of adaptation and acclimation of both host and microbial symbionts to environmental changes may provide leads to improve robustness of large‐scale cultivation of algae where environmental conditions cannot be as tightly controlled as in laboratory‐based experiments.

### Nutrient provision

Algae mainly need CO_2_ and inorganic sources of nitrogen and phosphate for growth along with some micronutrients and cofactors (Singh and Das, 2014). As fertilizer‐grade nutrient input accounts for a major proportion of cost in algal cultivation, recycling or provision of these nutrients via bacteria may eventually make large‐scale algal biomass production more economically viable (Clarens *et al*., 2010).

#### Macronutrients

CO_2_ is often the limiting substrate in large‐scale algal ponds because gas transfer efficiency is limited from ambient air (Putt *et al*., 2011). The main strategy to boost low CO_2_ concentrations in algal cultures is to use CO_2_‐enriched gases, but additional supply of CO_2_ comes with a significant cost (Clarens *et al*., 2010). Bacterial degradation of organic compounds released by algae contributes an additional source of CO_2_ for algal growth, especially during CO_2_‐limiting conditions as this CO_2_ can be fixed again by algae (Mouget *et al*., 1995; Subashchandrabose *et al*., 2011). This is exemplified with the case of a *Chlorella* sp. where carbon limitation was overcome when heterotrophic bacteria from a domestic wastewater treatment reactor were added to the algae culture and increased productivity of algal biomass by, respectively, 4.8‐ and 3.4‐fold in two independent experiments (Bai *et al*., 2015).

Nitrogen‐fixing bacteria reduce atmospheric dinitrogen to ammonium that is the major preferred nitrogen source for algae growth (Singh and Das, 2014). For example, *Bacillus pumilus* ES4 is a plant growth‐promoting bacterium that fixes nitrogen to enhance growth of *Chlorella vulgaris* (Hernandez *et al*., 2009). Symbiotic nitrogen fixers are also present in coral holobionts, where they co‐occur with *Symbiodinium* that is the most commonly coral‐associated dinoflagellate genus (Silverstein *et al*., 2012). Studies have revealed a strong positive correlation between the cell density of *Symbiodinium* and the number of nitrogen fixation gene copies from nitrogen‐fixing bacteria, which partly demonstrate how corals and their dinoflagellate partners could survive in low‐nutrient conditions (Reshef *et al*., 2006). The filamentous cyanobacteria *Richelia intracellularis* and *Calothrix rhizosoleniae* are close partners with diatoms living in the oligotrophic open ocean (Fiore *et al*., 2010). Higher growth rates were observed for diatoms with cyanobacteria as compared to diatoms without their nitrogen‐fixing cyanobacterial partners. Moreover, using single‐cell resolution analyses, it was shown that the N_2_ fixation rates of cyanobacteria increased by 171‐ to 420‐fold in symbiotic heterocystous cells associated with the corresponding diatoms as compared to free‐living cyanobacteria (Foster *et al*., 2011).

Phosphorus is an essential nutrient for algal growth. In most cases, algae can only take up inorganic phosphorus (*P*_i_) derived from hydrolysis of organic phosphorus (*P*_o_) (Zhu *et al*., 2013). Bacteria are the main agents involved in decomposing and mineralizing *P*_o_ through the secretion of phosphatases (Kononova and Nesmeyanova, 2002), and *P*_o_ from deteriorating algal cells can then be recycled to optimize algal yield on phosphate added. This process has been shown to occur with *Gordonia* sp. txj1302RI and *Burkholderia* sp. txj1302Y4, which degraded dissolved P_o_ to provide *Microcystis aeruginosa* with P_i_ needed for its growth in eutrophic lakes with abundant *P*_o_ but limited *P*_i_ (Zhao *et al*., 2012).

#### Vitamins, phytohormones, iron‐siderophore and antibiotics

Bacteria are not only capable of minimizing the requirement for external CO_2_ and major essential nutrients (N, P) for algae cultivation through regeneration or fixation (Reshef *et al*., 2006), but also provide algal hosts with vitamins (Croft *et al*., 2005; Grant *et al*., 2014), phytohormones (Amin *et al*., 2012, 2015; Sule and Belas, 2013; Segev *et al*., 2016), siderophores (Amin *et al*., 2009) and antibiotics (Seyedsayamdost *et al*., 2014). The heterotrophic bacterium *Dinoroseobacter shibae* DFL12^T^ has been demonstrated to provide growth‐limiting vitamins B_1_ and B_12_ to its dinoflagellate host. Based on a survey of 326 algal species, it was shown that vitamin B_12_ is required by more than half of the algal species (Croft *et al*., 2005). Epiphytic bacteria on seaweed (*Bacteroidetes* strain YM2‐23) produce the compound thallusin, which is essential for inducing growth, development and morphogenesis of *Monostroma oxyspermum* and other *Ulva* species (Matsuo *et al*., 2005; Twigg *et al*., 2014). *Sulfitobacter* sp. SA11 promotes diatom cell division via synthesis of the hormone indole‐3‐acetic acid (Amin *et al*., 2015). A *Marinobacter* sp. that lives in close association with *Scrippsiella trochoidea* is able to produce an unusual siderophore that promotes algal assimilation of iron (Amin *et al*., 2009). The marine bacterium *Phaeobacter gallaeciensis* produces growth hormones (phenylacetic acid) and a broad‐spectrum antibiotic (tropodithietic acid) against pathogenic bacteria, while the algal host (*Emiliania huxleyi*) provides fixed carbon in exchange (Seyedsayamdost *et al*., 2011).

Growing a particular strain of microalgae in an appropriate medium or adjusting media recipes for different algal growth stages remains a complicated task. In practice, most investigators tend to use a medium that works for their algae, but might not necessarily be the best one (Andersen, 2005). Understanding the symbiosis between microalgae and bacteria could lead to identification of missing medium components that could possibly be provided by cocultivation with bacteria.

## Harmful microbes in algal mass culture

One of the major risks of large‐scale intensive algae production is the emergence of viruses, parasites and bacterial pathogens (Pienkos and Darzins, 2009). Despite current advances in long‐term algae cultivation systems and farm management, it is neither cost‐effective nor achievable to completely avoid undesired contaminants at industrial scale (Cooper and Smith, 2015). An increasing number of pathogens and parasites have been discovered in recent years, and undoubtedly, this number will continue to grow as investment increases in algal farming (Hoffman *et al*., 2008; Georgianna and Mayfield, 2012).

**Figure 2:**
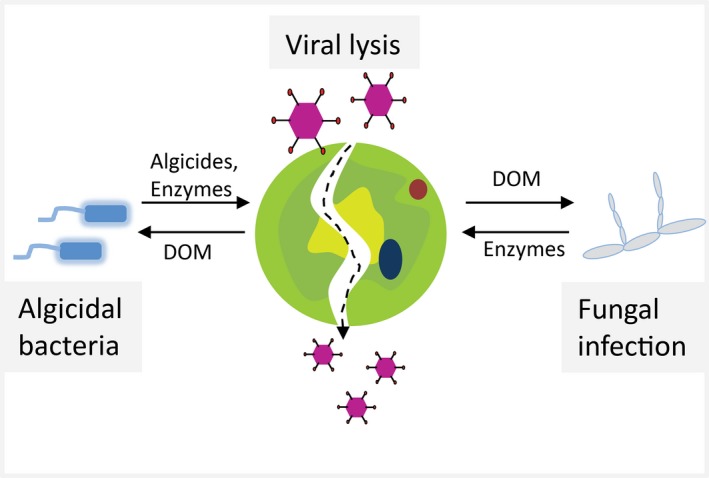
Illustration of antagonistic interactions between microalgae and microbes. DOM is dissolved organic matter.

As with terrestrial plants, algae are susceptible to infection by a wide range of viruses, bacteria, protists and fungi (Fig. 2; Carney and Lane, 2014). Oceanic algae are likely living with a multitude of viruses; however, only few algal viruses have been reported and characterized so far (Brussaard and Martinez, 2008). For example, the large double‐stranded DNA coccolithovirus (EhV, *Phycodnaviridae*) is able to terminate *Emiliania huxleyi* blooms (Wilson *et al*., 2002; Brussaard and Martinez, 2008; Schatz *et al*., 2014). Algae are also adversely affected by a wide range of bacteria; however, underlying mechanisms remain underexplored. Algae‐associated bacteria belonging to the families *Rhodobacteraceae*,* Saprospiraceae* and *Flavobacteriaceae* have been implicated in bleaching of the seaweed *Delisea pulchra* (Zozaya‐Valdés *et al*., 2017). Gram‐negative bacteria such as members of the genera *Alteromonas*,* Cytophaga*,* Flavobacterium*,* Pseudomonas*,* Saprospira*,* Vibrio* and *Pseudoalteromonas* are mainly responsible for rot symptoms (Ashen and Goff, 2000) and galls on seaweeds (Wang *et al*., 2008). Furthermore, *Microbacterium* sp. LB1 was shown to be responsible for algal cell lysis and damaged laboratory cultures of the green alga *Choricistis minor*, leading to dry weight reduction of 34% after 120 h of cultivation (Ivanova *et al*., 2014). Eukaryotic pathogens are prevalent but poorly understood, mostly because the strategies for detection, isolation and cultivation remain problematic (Gachon *et al*., 2010). A newly isolated algae‐lytic protist, *Pseudobodo* sp. KD51 the 18S rRNA gene of which shares 99% similarity with that of *Pseudobodo tremulans*, was shown to cause more than 50% decrease in chlorophyll content of *Chlorella vulgaris* after inoculation within three days. In addition to inhibition of *Chlorella vulgaris*,* Pseudobodo* sp. KD51 displayed a wide predatory spectrum and negatively affected the growth of *Dunaliella salina, Platymonas subcordiformis* and the cyanobacterium *Microcystis aeruginosa* (Chen *et al*., 2014). Rotifer grazers and ciliates prey on algal cells and can greatly decrease algal cell densities (Moreno‐Garrido and Canavate, 2001; Sarma *et al*., 2001). Fungi are known to parasitize microalgae and often caused lethal epidemics in algal cultures in which infection rates can reach 100% (Hoffman *et al*., 2008). So far, chytrid fungi have been reported to infect microalgae cultures of *Scenedesmus* (Carney *et al*., 2014), *Chlamydomonas* (Shin *et al*., 2001) and *Haematococcus pluvialis* (Hoffman *et al*., 2008).

### Identification and monitoring

Algal biomass losses due to contaminants such as chytrid parasites can be rapid (Carney *et al*., 2014). Therefore, fast and cost‐effective methods to identify and control potentially harmful organisms in algal production systems are necessary. However, microbial community composition in algal cultures is complex and dynamic. The composition may vary with location, cultivation cycle stage or method and season (Carney *et al*., 2014). Owing to the development of next‐generation sequencing methods, microbial identification can be carried out in a faster and less labour‐intensive way (Graham *et al*., 2015) and had been shown to effectively identify specific contaminants in algae cultivation reactors (Wichers *et al*., 2016) or toxic algal species (Edvardsen *et al*., 2013). When pond or photobioreactor performance is abnormal, a retrospective analysis of the archived samples could reveal harmful contaminants and inappropriate operation strategies. Knowledge from long‐term operation allows for identifying the most common and prevalent contaminants and this also gives operators predictive ability to some extent (Carney and Lane, 2014). Systematic analysis and characterization of contaminants can be used for the development of specific probes, primers or other biomarkers for rapid monitoring of algae production systems. For instance, before initiating large‐scale algae production, bacteria in algal inoculation stocks and the surrounding environments (water, soil, etc.) of the algae farm should be assayed for the presence of biological risks. A specific microbial pathogen library can be established and molecular tools can then be used to track harmful organisms of interest and improving cultivation management.

### Contamination and disease control

There is an increasing focus on preventing contamination to decrease major productivity losses in established systems (Stephens *et al*., 2010). Early detection and quantification of contaminants of algal cultures enable a fast response to infections. To protect algal cells from various contaminants, conventional methods such as physical filtration (Carney and Lane, 2014), applying decreased or elevated pH and temperatures (Borowitzka, 1999) and chemical agents (Lee, 2001) are neither effective nor economical in algal industry, and hence, new and efficient methods to combat contaminations are urgently needed.

*Phaeobacter inhibens* reciprocally exchange beneficial molecules with the microalga *Emiliania huxleyi*. Among these molecules is the antibiotic tropodithietic acid thought to kill other bacteria (Wang *et al*., 2016). In addition, a large screening of microbes indigenous to algae cultivation systems has led to the discovery of an antifungal protein produced by the bacterium *Streptomyces* sp. strain AP77. This protein has been used to cure red rot disease of *Porphyra* spp. seaweeds caused by *Pythium porphyrae* (Woo and Kamei, 2003). Hence, it is proposed that bacterial metabolites or bacteria that produce antimicrobial compounds could be supplied to bulk algal cultures in order to cost‐effectively achieve more robust cultures that are less prone to harmful invaders.

## Downstream processing of algal biomass using symbionts

Traditional mechanical or chemical pretreatment methods that are used to harvest algal biomass and disrupt algal cells require a large energy input and are cost‐intensive (Prajapati *et al*., 2015). To this end, algae‐associated microbes offer several new alternatives for microalgae harvest and cell wall disruption.

Harvesting algal biomass is one of most important economic factors in producing compounds with microalgae (Pienkos and Darzins, 2009). Harvesting algal cells is different from harvesting seeds of oil‐bearing plants, and oil extraction processes based on dry algal biomass are unlikely to be economical because of the high energy inputs needed to obtain dry algal biomass (Pienkos and Darzins, 2009; Ghasemi Naghdi *et al*., 2016). Currently, up to 50% of total cost of biodiesel production is spent on harvesting because of the high energy input and/or the addition of expensive chemicals. Energy‐intensive processes such as centrifugation are possible for high‐value products but are too costly for biofuel applications. In addition, other methods such as extensive use of chemical flocculants can be applied to aid in the harvesting process, but could only be cost‐effective when the required amount is small (Pienkos and Darzins, 2009). Therefore, development of economic and high‐efficiency harvesting techniques is important for alga bulk products, such as biofuels (Tanzi *et al*., 2013).

Bacteria can play an important role in microalgae aggregation (Grossart *et al*., 2006a,b). Diatom‐attached bacteria are capable of increasing diatom aggregate formation leading to the settling of photosynthetically active *Thalassiosira weissflogii*, while free‐living bacteria are not involved in this process (Gärdes *et al*., 2011). In another study, mass cultures of *Nannochloropsis* were observed to form aggregates that consisted of algal cells, bacteria and debris that together resulted in a complex structure (Rodolfi *et al*., 2003). Wang *et al*. isolated a novel bacterium HW001 from Permian groundwater and demonstrated that this strain is able to stimulate aggregation of both *Nannochloropsis oceanica* IMET1 and other potential biofuel‐producing green microalgae, diatoms and cyanobacteria (Wang *et al*., 2012a). In addition, two potent bioflocculants have been discovered from culture supernatant of *Burkholderia cepacia* (Manheim and Nelson, 2013) and *Bacillus licheniformis* CGMCC 2876 (Ndikubwimana *et al*., 2016). High flocculation efficiency of *Desmodesmus brasiliensis* (> 98 %) was achieved at pilot‐scale treatment with poly‐γ‐glutamic acid, a bioflocculant produced by *Bacillus licheniformis* CGMCC 2876 (Manheim and Nelson, 2013).

Besides bacteria, a number of filamentous fungal strains have also been reported to promote flocculation of microalgae (Zhang and Hu, 2012; Xie *et al*., 2013a, b; Wrede *et al*., 2014). Muradov *et al*. tested the fungal species (*Aspergillus fumigatus*) in co‐culture with freshwater and seawater algal species and showed up to 90% flocculation after 24 h of cultivation, while no aggregates were formed in the absence of the fungus. Furthermore, algal–fungal copelletization improved oil extraction efficiency because fungal secreted hydrolytic enzymes disrupted the thick cell walls of *Tetraselmis suecica* (Muradov *et al*., 2015). The same was seen between *Aspergillus lentulus* FJ172995 and *Chroococcus* sp., where algal and fungal cells formed a pellet, and nearly 100% of biomass settled down within 6 h at an optimized fungal/algal ratio of 1:3 (Prajapati *et al*., 2016).

## Algae–bacteria‐based wastewater treatment

High biomass production costs obstruct the economic feasibility and competitiveness of algal biofuels (Olguín, 2012). The application of a combination of algae cultivation and wastewater treatment could provide a win‐win solution to this problem (Pienkos and Darzins, 2009; Unnithan *et al*., 2014). Wastewater from municipal sources, pig production, aquaculture and dairy cattle farming is rich in nutrients such as nitrates, ammonia and phosphates, which can be used for algae cultivation (Singh and Das, 2014). Mixed algal–bacterial populations in wastewater can not only perform more diverse tasks than single strains but are also better equipped to tolerate environmental fluctuations and pathogen invasions (Subashchandrabose *et al*., 2011). Moreover, the combination of algae and bacteria improves water treatment efficiency, and simultaneously, the harvested algal biomass as by‐product has been considered a promising source for feeds, biofuels and fertilizer (Azim and Little, 2008; Unnithan *et al*., 2014).

### Carbon, nitrogen and phosphate removal

Algae produce oxygen during photosynthesis that is used by bacteria to mineralize organic matter (Guieysse *et al*., 2002). Carbon dioxide released by bacteria during mineralization can in turn be utilized by algae (Munoz and Guieysse, 2006). Concurrently, abundant compounds in wastewater, such as ammonium and phosphate are eliminated by algal uptake (Wang and Lan, 2011). Su *et al*. noted that the synergistic cooperation between photosynthetic organisms, including algae and cyanobacteria, and activated sludge bacteria enhanced organic carbon removal efficiencies (Su *et al*., 2012). More than 91.2% of chemical oxygen demand was removed, and the highest total nitrogen and phosphorus removal rates were 91.0 ± 7.0% and 93.5 ± 2.5% respectively. *Chlorella sorokiniana* (González *et al*., 2008) and *Euglena viridis* (de Godos *et al*., 2010) were also shown to enhance removal of carbon, nitrogen and phosphorous from piggery waste water when mixed with bacteria from activated sludge.

### Removal of heavy metals and toxic organic compounds

In addition to enhanced removal of excessive nutrients, algal–bacterial consortia were also shown to be capable of removing heavy metals and toxic organic compounds from wastewater (Munoz and Guieysse, 2006). Algal cells not only provide stable habitats for the bacteria but also concentrate pollutants to enhance bioavailability for bacterial degradation (Gutierrez *et al*., 2014). Algal–bacterial consortia successfully achieved higher biodegradation or removal rates of pollutants than single species (Luo *et al*., 2014).

Heavy metals belong to an important group of contaminants that pose global environmental risks (Järup, 2003). Co‐cultures of bacteria and algae were capable of removing 80% of the copper and 100% of the cadmium from wastewater in a continuous flow‐through column (Subashchandrabose *et al*., 2011). In addition, a biofilm with immobilized algae (*Ulothrix* sp.) and bacteria in a photo‐rotating biological contactor removed 20‐50% of a large variety of metals (Cu > Ni > Mn > Zn > Sb > Se > Co > Al) within a 10‐week period (Orandi *et al*., 2012).

Polycyclic aromatic hydrocarbons are ubiquitous pollutants in various niches that might cast high risks on human and animal health (Wang *et al*., 2012a, b). A co‐culture of the alga *Chlorella sorokiniana* and *Pseudomonas migulae* demonstrated higher phenanthrene degradation rates than most of the values reported in the literature (Muñoz *et al*., 2003). Luo *et al*. established a consortium consisting of microalgae (*Selenastrum capricornutum*) and a bacterium (*Mycobacterium* sp. strain A1‐PYR) that achieved faster degradation of pyrene than the systems that used algae or bacteria alone (Luo *et al*., 2014). The same result was obtained by a synthetic consortium combining *Synechocystis* sp. and pyrene‐degrading bacteria (*Pseudomonas* sp. and *Bacillus* sp.). The combination increased both algal growth and degradation of the polycyclic aromatic hydrocarbon (Patel *et al*., 2015).

Given the abovementioned advantages, integration of algae and bacteria has a large potential for wastewater treatment, especially under aerobic conditions. Oxygen produced by algae in the system can reduce the aeration demand in conventional activated sludge systems, which accounts for nearly 50% of the total energy input of the water treatment plants (Rawat *et al*., 2011). In addition, removing nutrients from wastewater with a combination of algae and bacteria can increase the removal efficiency, system robustness and application potential of the sludge.

## Outlook

Unravelling the complex biological mechanisms of algal–microbial interactions represents a largely understudied realm to improve production of high‐value products and biofuels through large‐scale cultivation of microalgae. Protective bacteria could inhibit growth of bacterial or fungal contaminants, which cause fouling or negatively affect algal growth. Macrofertilizers and expensive micronutrients supplied by bacterial metabolism can reduce the need for external input. Some bacteria are able to enhance synthesis of desired algal metabolites, for instance, lipids. However, currently our knowledge on algae–bacteria interactions is too scattered to identify generalities with respect to bacterial species that are suitable for co‐culture with microalgae. Alga species‐specific knowledge would logically be first developed for industrial working horse species, such as *Arthrospira* spp., *Chlorella* spp., *Scenedesmus* spp., *Nannochloropsis* spp. and *Botryococcus* spp.(Mobin and Alam, 2017). In addition, the desired microbial community in algae cultures may depend on the required product specifications (biofuel, feed and food and fine chemicals) and harvesting methods applied.

**Figure 3:**
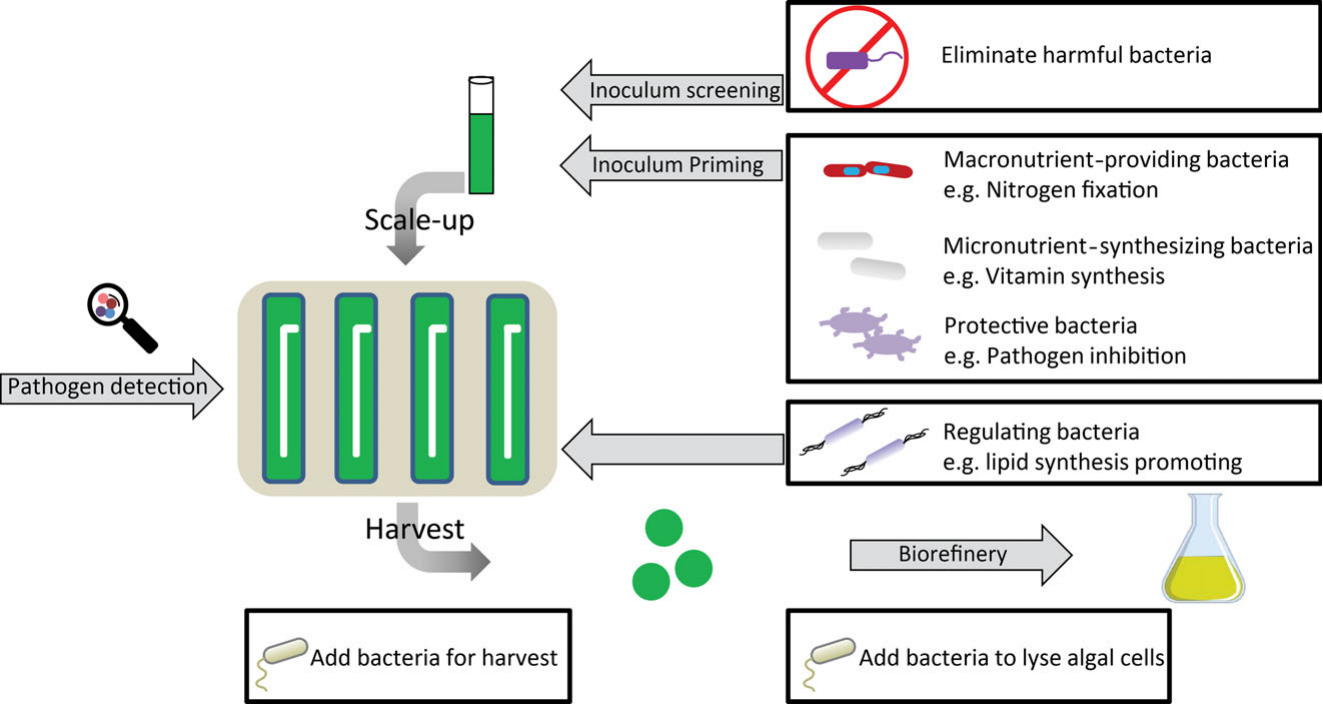
Potential integration strategies for including microbial community management into photobioreactor operations.

Further insights into evolution and establishment of mutualistic interactions allow for developing more resilient synthetic co‐cultures (Fig. 3). Real‐time monitoring techniques are important to maintain stable and healthy mixed cultures in outdoor ponds exposed to changing weather and ubiquitous invaders. The main challenges for the application of bacteria in algal cultivation are to steer the bacterial community to its desired composition and how to maintain this balance during different modes of operation, different reactor types and fluctuations in outdoor conditions. The establishment and maintenance of optimized algae–bacterial co‐cultures may require bioreactor operation management strategies that are extended beyond the performance of microalgae in the system, but consider and value the community present as a whole.

## Conflict of interest

The authors declare no conflict of interest.
